# EGFR/Ras-induced CCL20 production modulates the tumour microenvironment

**DOI:** 10.1038/s41416-020-0943-2

**Published:** 2020-06-30

**Authors:** Andreas Hippe, Stephan Alexander Braun, Péter Oláh, Peter Arne Gerber, Anne Schorr, Stephan Seeliger, Stephanie Holtz, Katharina Jannasch, Andor Pivarcsi, Bettina Buhren, Holger Schrumpf, Andreas Kislat, Erich Bünemann, Martin Steinhoff, Jens Fischer, Sérgio A. Lira, Petra Boukamp, Peter Hevezi, Nikolas Hendrik Stoecklein, Thomas Hoffmann, Frauke Alves, Jonathan Sleeman, Thomas Bauer, Jörg Klufa, Nicole Amberg, Maria Sibilia, Albert Zlotnik, Anja Müller-Homey, Bernhard Homey

**Affiliations:** 1 Department of Dermatology, Medical Faculty, Heinrich Heine University, Düsseldorf, Germany; 2 Department of Dermatology, University Hospital Münster, Münster, Germany; 3 Department of Dermatology, Venereology and Oncodermatology, Medical Faculty, University of Pécs, Pécs, Hungary; 4 Department of Pediatric Cardiology and Intensive Care Medicine, Medical Center Georg August University Göttingen, Göttingen, Germany; 5 Clinic of Hematology and Medical Oncology, University Medical Center Göttingen, Göttingen, Germany; 6 Dermatology and Venereology Unit, Department of Medicine, Karolinska Institutet, Stockholm, Sweden; 7 Department of Dermatology and UCD Charles Institute for Dermatology, University College Dublin, Dublin, Ireland; 8 Department of Pharmacology, Medical Faculty, Heinrich Heine University, Düsseldorf, Germany; 9 Precision Immunology Institute, Icahn School of Medicine at Mount Sinai, New York, NY USA; 10 Leibnitz Research Institute for Environmental Medicine, Düsseldorf, Germany; 11 Department of Physiology and Biophysics, University of California, Irvine, CA USA; 12 Department of General-, Visceral- and Pediatric Surgery, Medical Faculty, Heinrich Heine University, Düsseldorf, Germany; 13 Department of Otorhinolaryngology, Head and Neck Surgery, University Medical Center, Ulm, Germany; 14 European Center for Angioscience (ECAS), Medical Faculty of Mannheim, Heidelberg University, Mannheim, Germany; 15 Karlsruhe Institute for Technology (KIT), Campus Nord, Institute for Toxicology and Genetics, Karlsruhe, Germany; 16 Institute of Cancer Research, Department of Medicine I, Medical University of Vienna and Comprehensive Cancer Center, Vienna, Austria; 17 Insitute of Science and Technology Austria, Klosterneuburg, Austria; 18 Insitute for Immunology, University of California, Irvine, CA USA; 19 Department of Radiation Oncology, Medical Faculty, Heinrich Heine University, Düsseldorf, Germany

**Keywords:** Cancer microenvironment, Chemokines

## Abstract

**Background:**

The activation of the EGFR/Ras-signalling pathway in tumour cells induces a distinct chemokine repertoire, which in turn modulates the tumour microenvironment.

**Methods:**

The effects of EGFR/Ras on the expression and translation of CCL20 were analysed in a large set of epithelial cancer cell lines and tumour tissues by RT-qPCR and ELISA in vitro. CCL20 production was verified by immunohistochemistry in different tumour tissues and correlated with clinical data. The effects of CCL20 on endothelial cell migration and tumour-associated vascularisation were comprehensively analysed with chemotaxis assays in vitro and in CCR6-deficient mice in vivo.

**Results:**

Tumours facilitate progression by the EGFR/Ras-induced production of CCL20. Expression of the chemokine CCL20 in tumours correlates with advanced tumour stage, increased lymph node metastasis and decreased survival in patients. Microvascular endothelial cells abundantly express the specific CCL20 receptor CCR6. CCR6 signalling in endothelial cells induces angiogenesis. CCR6-deficient mice show significantly decreased tumour growth and tumour-associated vascularisation. The observed phenotype is dependent on CCR6 deficiency in stromal cells but not within the immune system.

**Conclusion:**

We propose that the chemokine axis CCL20–CCR6 represents a novel and promising target to interfere with the tumour microenvironment, and opens an innovative multimodal strategy for cancer therapy.

## Background

Tumours arise from normal cells through genetic alterations that affect the tight regulation of growth control. During tumorigenesis, continuous paracrine communication between the tumour cells and the surrounding microenvironment creates a dynamic signalling circuitry that promotes cancer initiation, growth and metastasis, resulting ultimately in the demise of the patient.^1^ The tumour microenvironment is composed of distinct cell types, including endothelial cells, pericytes, fibroblasts as well as resident and infiltrating leukocyte subsets.^2^ Changes in the microenvironment are initiated, in part, by the activation of growth factor receptors. In particular, the epidermal growth factor receptor (EGFR) is overexpressed and/or activated in a wide variety of cancers, as is the mitogen-activated protein kinase (MAPK) signalling cascade Ras/Raf/MEK/ERK.^3^ Uncontrolled activation of the EGFR/Ras-signalling pathway is, therefore, an important step towards carcinogenesis and the co-evolution of a tumour-supporting microenvironment.

Notably, transformed epithelial cells overexpress a distinct set of chemokine receptors, and also modulate chemokine production by EGFR/Ras activation when compared with benign precursor cells. This contributes to the establishment of a tumour-promoting microenvironment that facilitates tumour-associated angiogenesis and metastasis.^4,5^

Previously, we demonstrated that activation of EGFR/Ras led to the downregulation of the homoeostatic chemokine CCL27 in transformed keratinocytes, resulting in significantly impaired recruitment of tumour-targeting leukocytes, leading to evasion from tumour-immune surveillance.^6^

Here we report that the chemokine CCL20 is highly upregulated in transformed keratinocytes after activation of the EGFR/Ras-signalling pathway. CCL20 expression is also upregulated in tumour cells isolated from melanoma, breast cancer, colon carcinoma and head and neck squamous cell carcinoma (HNSCC) patients, in conjunction with activation of the EGFR/Ras-signalling pathway. In addition, tumour progression and poor survival of breast cancer patients correlate with CCL20 expression. At the cellular level, we find that CCL20 directly targets endothelial cells, both in vitro and in vivo, via its specific cognate receptor CCR6. Activation of CCR6 signalling in endothelial cells induces cell migration and enhances vessel formation in vitro and in vivo. Furthermore, tumour growth and vascularisation of B16F10-derived murine melanoma was dramatically inhibited in CCR6-deficient C57BL/6 mice in comparison with wild-type C57BL/6 mice. Moreover, experiments with bone marrow chimeras indicate that tumour-promoting effects of CCR6 signalling are dependent on CCR6^+^ stromal but not on CCR6^+^ immune cells. We, therefore, propose that the chemokine axis CCL20–CCR6 represents a novel and promising target to interfere with the tumour microenvironment, and opens an innovative multimodal strategy for cancer therapy.

## Methods

### Biopsy samples

Six-millimetre punch biopsies were taken after obtaining informed patient consent. Biopsies were acquired from breast cancer patients, HNSCC and melanoma patients. Normal skin and normal breast samples were obtained from individuals undergoing plastic surgery. Tumour tissue microarrays of breast cancer and head and neck squamous cell carcinoma were acquired from Pantomics, Inc. (San Francisco, CA, USA), Super Bio Chips (Seoul, Korea) and US Biomax, Inc. (Rockville, MD, USA). Colon carcinoma tumour tissue microarrays were provided by N. Stoecklein (Department of General-, Visceral- and Pediatric Surgery, University Hospital Düsseldorf, Germany). All studies were approved by the appropriate ethics committees.

### Mice

Wild-type C57BL/6 mice were purchased from Taconic, Denmark. C57BL/6–CCR6^−/−^ mice^7^ were a kind gift from S.A. Lira (Immunobiology Center, Mount Sinai School of Medicine, New York, NY, USA). Mice had a median age of 10 weeks. Mice were housed in the animal facility of the Heinrich-Heine-University Düsseldorf, Germany. Experiments were approved by the animal ethics committee of the “Bezirksregierung Düsseldorf” based on the German “Tierschutzgesetz”. For the Matrigel plug assays, syngeneic skin tumour model and bone marrow chimera-only male mice were used in the experiments. EGFRfl/fl mice,^8^ K5Cre transgenic mice^9^ and *K5-SOS* transgenic mice^10^ were kept in the animal facilities of the Medical University of Vienna in accordance with institutional policies and federal guidelines. Animal experiments were approved by the Animal Experimental Ethics Committee of the Medical University of Vienna and the Austrian Federal Ministry of Science and Research (animal licence numbers: GZ 66.009/124-BrGT/2003; GZ 66.009/109-BrGT/2003; BMWF-66.009/0073-II/10b/2010; BMWF-66.009/0074- II/10b/2010). EGFRfl/fl mice were kept in C57BL/6 background for more than 30 generations, and were crossed with K5Cre transgenic mice. No phenotypic differences have been identified between male and female mice; thus, all experiments were conducted using both genders. All mice were kept in cages with own ventilation, and had access to food and water ad libitum. All mice were sacrificed directly from their home cage by cervical dislocation in the afternoon, and skin samples were taken immediately thereafter and stored in liquid nitrogen for further analysis.

### Cell culture

Human primary epidermal keratinocytes were purchased from Cambrex (East Rutherford, NJ, USA) and cultured as described.^11^ Cells were treated with 500 nM or 1 µM erlotinib (Roche, Mannheim, Germany) for 24 h or left untreated. HaCaT cells were transfected with an activating mutant of Ras (H-RasV12) in the expression vector pDCR (a gift from Craig Webb, Frederick, MD) as described by Tscharntke et al.^12^ HaCaT keratinocytes and H-RasV12-transfected HaCaT clones were cultured as described previously.^13^ The following human breast cancer (MDA-MB-468, DU-4475, MCF-7, T-47D, Hs578T and MDA-MB-361) and melanoma cell lines (MDA-MB-435S, MeWo, SK-Mel-2, SK-Mel-28, SK-Mel-31, HT-144, Malme-3M and B16F10) were obtained from the ATCC and cultured as recommended by the provider. MV3 and BLM melanoma cells were a gift from D. J. Ruiter (Department of Pathology, University Medical Center Nijmegen, The Netherlands). Cells were cultured in DMEM (Lonza, Basel, Switzerland) supplemented with 10% FCS (Biochrom AG, Berlin, Germany). TXM-13 and TXM-40 melanoma cells were obtained from I.J. Fidler (Department of Cancer Biology, Cancer Metastasis Research Center, Houston, TX, USA) and cultured as described.^14^ UKRV-Mel-4 and UKRV-Mel-30 melanoma cells were a gift from D. Schadendorf (Department of Dermatology, Venereology and Allergology, University Medical Center and Medical Faculty Mannheim, University of Heidelberg, Mannheim, Germany) and grown in RPMI-1640 medium (Lonza) supplemented with 10% FCS (Biochrom AG). UD-SCC squamous cancer cell lines were derived from primary tumours of the head and neck region, and UM-SCC cell lines were generated from lymph node metastases by the Institute of Otorhinolaryngology of the University Hospital Düsseldorf, Germany. These cells were cultured in DMEM (Lonza) with 10% FCS (Biochrom AG) (UD-SCC 1–2, 4, 7–8, UM-SCC 10B, 17B, 24A and 24B) or in MEM (Gibco, Eggenstein, Germany) with 10% FCS and 2% L-glutamine (PAA, Pasching, Austria) (UD-SCC 3, 6, UM-SCC 10A, 17A). Human primary mammary epithelial and human primary melanocytes were obtained from Clonetics (San Diego, CA, USA) and cultivated in MEGM complete medium and MGM-4 medium (all Lonza). Human primary mucosal keratinocytes were generated from punch biopsies and cultured similarly to the primary human keratinocytes. Human dermal microvascular blood endothelial cells (BEC) and human lymphatic endothelial cells (LEC) were cultured in endothelial cell growth medium (EGM-2) and purchased from Lonza. All cells were routinely cultured in an incubator at 37 °C with 95% humidity and 5% CO_2_ (INCO 2, Memmert, Schwabach, Germany), and all media were supplemented with 1% of a mixture of antibiotics (penicillin 100 U/ml, streptomycin 100 μg/ml) (PAA).

### Ras activity assay

The activity of H-Ras proteins was determined by using the Active Ras Pull-Down and Detection Kit as described by the manufacturer (ThermoFisher Scientific Pierce Protein Research Products, Waltham, MA, USA).

### Quantitative real-time RT-PCR (TaqMan) analysis

Quantitative real-time RT-PCR analysis was performed as previously described.^13^ Briefly, after isolation of total RNA from skin biopsies by TRIzol (Invitrogen, Karlsruhe, Germany) extraction, 4 µg of total RNA was treated with 10 U of DNase I (Roche) and reverse-transcribed into cDNA. About 25 ng of cDNA was amplified in a volume of 25 µl in the presence of TaqMan universal master mix (Applied Biosystems, Darmstadt, Germany), 0.3 µM gene-specific TaqMan probe and 0.24 µM gene-specific forward and reverse primers. Primers and target-specific probes were obtained from Applied Biosystems, and gene-specific PCR products were measured by means of an ABI PRISM 7000 (Applied Biosystems) during 40 cycles. Target gene expression was normalised to the expression of 18 S rRNA.

### Immunohistochemistry

Frozen and paraffin-embedded tumour sections from cancer patients were stained with antibodies against human CCL20 (goat IgG: R&D Systems, Minneapolis, MN, USA; horse IgG: Vector Laboratory’s Vectastain ABC peroxidase kit) or human pERK (mouse IgG1: Cell Signaling, Danvers, MA, USA; horse IgG: Vector Laboratory’s Vectastain ABC peroxidase kit). Frozen matrigel plugs were cut into 8-µm tissue sections and fixed in acetone before immunostaining. Sections were pretreated with H_2_O_2_ and stained with an antibody against murine CD31 (rat IgG2a: Acris Antibodies, Herford, Germany; Vector Laboratory’s AEC peroxidase kit, Burlingame, CA, USA). Paraffin-embedded mouse tumours were sectioned and stained with antibodies against murine CD31 (rat IgG2a: Acris Antibodies; Linaris Histoprime’s HistoGreen Substrate Kit for Peroxidase). Images were acquired using a Zeiss Cell Observer (Zeiss Axiovision 4.6 software, Carl Zeiss Microimaging, Göttingen, Germany).

### Immunofluorescence

BECs and LECs were fixed in paraformaldehyde and stained against human CCR6 (mouse IgG1: BD Pharmingen, San Diego, CA, USA). Frozen 8-µm tumour tissue sections were fixed in paraformaldehyde and stained with antibodies against human CCR6 (mouse IgG2b: R&D Systems) and human CD31 (mouse IgG2a: BD Pharmingen). All cells were additionally stained with DAPI (Invitrogen).

### In vitro monolayer wound-healing assay

BECs were cultured to confluence in 24-well plates. Scratch wounds were introduced in the monolayers by using a sterile 0.1–10-μl pipette tip. After washing away cells in suspension, cultures were supplied with cell medium containing 0 or 100 ng/ml recombinant CCL20 (R&D Systems). Imaging was performed with a Zeiss Cell Observer (Zeiss).

### Tube-formation assay

In total, 2 × 10^3^ BECs were seeded in µ-slide angiogenesis chambers (Ibidi, Munich, Germany) on growth factor-reduced matrigel (BD Biosciences, San Jose, CA, USA). Cells were supplemented with 0, 10, 100 or 1000 ng/ml of recombinant CCL20 (R&D Systems) in EGM-2MV containing 5% FCS (Clonetics). Tube formation was imaged with a Zeiss Cell Observer (Zeiss).

### Chemotactic cell migration

Chemotactic cell migration assays were performed according to the µ-slides chemotaxis manual (Ibidi) using collagen-coated chemotaxis µ slides. BECs were seeded into observation chambers at a density of 1 × 10^6^ cells/ml. CCL20 (R&D Systems) and CCL21 (R&D Systems) gradients were generated at 1000–10 ng/ml. In controls, cells were pretreated with 30 µg/ml anti-human CCR6 antibody (R&D Systems) for 10 min, and anti-human CCR6 antibody was also included during the experiment. Cells were observed for 60 h with a Zeiss Cell Observer (Zeiss). Data were analysed using ImageJ 1.37c software (Wayne Rasband, National Institute of Health, Bethesda, MD, USA).

### ELISA

Quantification of CCL20 concentration in cell culture supernatants was performed according to the DuoSet ELISA Development kit protocol (R&D Systems).

### Flow cytometric analysis of CCR6 expression

BECs and LECs were analysed using flow cytometry. Briefly, 10^6^ cells were stained with PE-labelled anti-human CCR6 antibodies (mouse IgG1: BD Pharmingen) and anti-human Podoplanin antibodies (mouse IgG1, clone D2-40: Dako, Glostrup, Denmark). Cells were fixed in 1% paraformaldehyde and then analysed with a FACSCalibur and CellQuest software (BD Biosciences).

### Matrigel plug assay

Male wild-type C57BL/6 mice (*n* = 6) and C57BL/6–CCR6^−/−^ mice (*n* = 6) were injected subcutaneously in the lateral flanks with 0.5 ml of pure matrigel (BD Biosciences) or matrigel containing 1000 ng/ml recombinant murine CCL20 or 1000 ng/ml recombinant human CCL21 (all R&D Systems). After 21 days, mice were sacrificed by cervical dislocation; Matrigel plugs were excised and frozen. Neovascularisation in the plugs was analysed by immunohistochemistry.

### Murine syngeneic skin tumour model B16/F10

B16/F10 cells (2.5 × 10^5^) in 100 µl of PBS were injected subcutaneously into the lateral right flank of male wild-type C57BL/6 mice (*n* = 12) and C57BL/6–CCR6^−/−^ mice (*n* = 12) during the morning in the animal housing facility. After tumour growth for 14 days, mice were imaged with a fpVCT, a nonclinical volume CT prototype (GE Global Research, Niskayuna, NY, USA) as previously described.^15^ Mice were transported to the fpVCT facility at least 2 days before experimentation. In the morning on the day of the experiment, mice were anaesthetised with vaporised isoflurane at 0.8–1% concentration throughout the imaging session. The iodine-containing contrast agent, Ultravist 150 (at 150 µl per mouse, Bayer-Schering Pharma AG, Berlin, Germany), was applied intravenously ~30 s before the scan. At the end of the experiment in the early afternoon, mice were sacrificed by cervical dislocation, autopsied and tumours were excised, weighed, paraffin-embedded and analysed by immunohistochemistry.

### Generation of mouse bone marrow chimeras

Recipient male wild-type C57BL/6 mice and C57BL/6–CCR6^−/−^ mice were kept under antibiosis in drinking water (100 mg/l Ciprofloxacin (Ciprobay®, Bayer Vital GmbH)) for 7 days before irradiation. Generation of chimera was performed by irradiation of the mice with 8 Gray (Gy) using a Co^60^-radiation source to destroy the bone marrow of the mice. Mice were rehoused in the animal facility under sterile conditions with a closed ventilation system. On the next day, donor mice were sacrificed by manual cervical dislocation and then shortly bathed in 70% ethanol (Merck, Darmstadt, Germany) before preparation of the femur and the calf. Both were placed in a small petri dish with ice-cold PBS (PAA, Pasching, Austria), and afterwards condyles were cut off on both sides, and the bone marrow was rinsed out of the bone with ice-cold PBS (PAA, Pasching, Austria) by using a syringe and a needle. In total, 1 million cells per 250 µl of PBS (PAA, Pasching, Austria) were injected into the tail vein of recipient, irradiated mice. The following combinations of chimera were generated: mice with a *Ccr6*^*−/*−^-deficient stroma and a wild-type immune system (*Ccr6*^−*/−*^ → *WT*), wild-type mice bearing a wild-type immune system (*WT* → *WT*) and wild-type mice with a *Ccr6*^*–/–*^ immune system (*WT* → *Ccr6*^*−/−*^). After bone marrow transplantation, mice were observed for another 6 weeks under Ciprofloxacin antibiosis and weighed three times a week on non-consecutive days to check their state of health. Mice were observed for another 7 days without antibiosis before injection with B16/F10 cells.

### Statistical analysis

Statistical analyses were calculated using the nonparametric Mann–Whitney *U* test. To test the correlation between the clinicopathological data and the level of CCL20 expression, as well as the correlation between pERK and CCL20 expression, we used Fisher’s exact test and whenever appropriate logistic regression. All of the variables were dichotomised. For analysis of follow-up data, lifetable curves were calculated using the Kaplan–Meier method, and survival distributions were compared using the log-rank test. The primary endpoints were disease-specific or relapse-free survival, as measured from the date of surgery to the time of the last follow-up or cancer-related death. Variations were considered to be statistically significant at values of *p* < 0.05. *P* values < 0.05 are indicated by *, <0.01 by ** and *p* values < 0.001 by ***. The number of animals was kept small according to the expected differences. All animals survived and were included in statistical analysis.

### Publicly available datasets

All publicly available data used in the paper were obtained from the NCBI Gene Expression Omnibus (GEO) Dataset collection in the form of prefiltered, normalised expression tables found under the respective accession numbers: GDS5085, GSE29415, GSE12445 and GSE115978. Differential gene expression was tested using linear model fitting followed by Benjamini–Hochberg multiple testing correction. Graphs were generated using Graphpad Prism v.8 software.

### Flow cytometry of murine keratinocytes

Epidermal cell suspensions were generated by surgically removing the trunk skin and gently scraping off subcutaneous adipose tissue using a scalpel knife. Skin was then placed dermal side down on DMEM (Gibco) containing 2 mM EDTA and 0.25% Trypsin (Gibco) at 4 °C overnight. The next day, the epidermis was softly scraped off and further dissociated into single cells by pipetting up and down after adding DMEM containing 2% chelexed FCS (Sigma). Cells were filtered using 70-μm and 40-μm cell strainers (BD), centrifuged at 300 × *g* for 10 min and washed with PBS (Sigma) containing 2% chelexed FCS twice before staining. Epidermal single-cell suspensions were incubated with the following antibodies on ice for 30 min: a6-PE (BD, 1:50), Scal-PECy7 (Biolegend, 1:100) and CD45-APCCy7 (BD, 1:200). Dead cell exclusion was performed using Hoechst (1 μg/ml). Cell sorting of ~8,000 keratinocytes derived from interfollicular epidermis (IFE) (characterised as a6 high, ScaI high) from individual P2 pups was performed using a BD AriaI and BD FACSDiva software.

### RNA isolation and reverse transcription

Approximately 8000 IFE keratinocytes were directly sorted into RNA Extraction Buffer of the PicoPure RNA Isolation Kit (Arcturus), incubated at 42 °C for 30 min and centrifuged at 800 × *g* for 2 min. Supernatants were transferred into new tubes and stored at −80 °C until further RNA purification according to the manufacturer’s instructions, despite the last step. For RNA elution, we did not use the provided Elution Buffer, but RNase-free water in order to be able to measure RNA quality with the BioAnalyzer (Total Eukaryote RNA Pico Kit, Agilent, according to the manufacturer’s instructions). About 1 μl of RNA was used to generate cDNA using VILO cDNA synthesis kit (Life Technologies) according to the manufacturer’s protocol. cDNA was stored at –20 °C until further use.

## Results

### The EGFR/Ras-signalling pathway regulates chemokine production

The Ras–MAPK pathway is frequently deregulated in tumours arising from epithelial tissues (e.g. carcinomas and melanoma) as a result of genetic alterations and upstream activation of cell surface growth factor receptors (e.g. EGFR). To identify chemokines that are regulated by oncogenic Ras activation in epithelial cells, we systematically analysed chemokine expression in HaCaT keratinocytes transfected with the H-RasV12 oncogene (Fig. S1). We identified two distinct Ras-dependent regulation patterns of chemokine production. The first set of epithelial cell-associated chemokines, represented by CXCL14 and CCL27, was markedly downregulated following transfection with oncogenic Ras confirming previous results^6^ (Fig. S1B, C). Second, a set of chemokines represented by CCL20 and CXCL8 was markedly induced through activation of the Ras-signalling pathway (Fig. S1D, E). Since CCL20 showed the most prominent Ras-dependent upregulation among all tested chemokines, and previous findings supported a role for CXCL14 and CCL27 in tumour-immune surveillance, we decided to investigate further the role of CCL20 within the tumour microenvironment.

### EGFR/Ras activation enhances CCL20 production

**Fig. 1:**
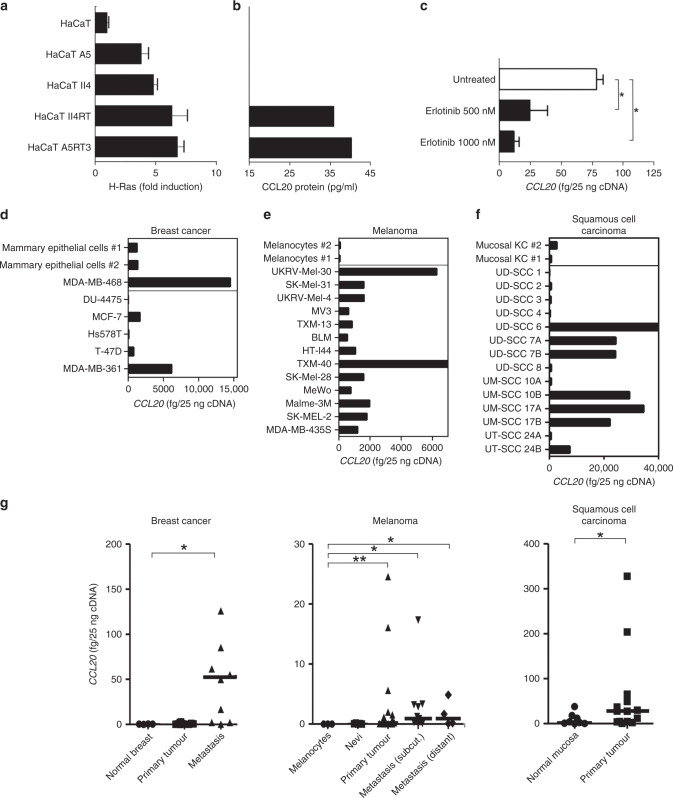
Oncogenic Ras induces CCL20 protein synthesis. **a** Relative Ras activity in the immortalised keratinocyte cell line HaCaT and in H-RasV12-transfected HaCaT clones was assayed by the EZ-Detect Ras activation kit. **b** CCL20 protein production- conditioned medium from HaCaT cells and H-RasV12-transfected HaCaT clones, as detected by a CCL20-specific ELISA. **c** Activated primary keratinocytes were treated with the selective irreversible inhibitor of EGFR erlotinib, and expression of CCL20 was analysed by qPCR. Tumour cells derived from breast cancer, malignant melanoma and head and neck squamous cell carcinoma (HNSCC) overexpress CCL20. **d**–**f** Quantitative real-time PCR analysis of CCL20 in cultured normal primary mammary epithelial cells (*n* = 2) and breast cancer cell lines (*n* = 7) (**d**), cultured normal primary melanocytes (*n* = 2) and melanoma cell lines (*n* = 13) (**e**), cultured primary mucosal keratinocytes (KC, *n* = 2), cell lines derived from primary tumours (*n* = 10) or metastases (*n* = 4) of HNSCC (**f**). **g** CCL20 expression of tumour tissues derived from breast cancer (primary breast cancer, *n* = 12, fc = 1.096; breast cancer metastasis, *n* = 10, fc = 6.048), malignant melanoma (primary melanoma, *n* = 28, fc = 4.580; subcutaneous metastasis, *n* = 11, fc = 6.023; distant metastasis, *n* = 4, fc = 6.012) and HNSCC (primary tumour, *n* = 14, fc = 3.057) compared with normal tissue (normal breast, *n* = 3, cultured primary melanocytes, *n* = 3, benign nevi, *n* = 5 and normal oral mucosa, *n* = 8) by qPCR. Values are either expressed as femtograms of target gene per 25 ng of cDNA or protein concentration in picograms per ml of supernatant and represent the mean ± SD of three independent experiments (*P ≤ 0.05; **P ≤ 0.01; Mann–Whitney *U* test).

To further examine H-RasV12-transfected HaCaT keratinocyte-associated CCL20 induction, we measured CCL20 secretion into cell culture supernatants of HaCaT H-RasV12 clones using ELISA, and found detectable levels of CCL20 protein in the HaCaT clones with high Ras activity (HaCaT II4RT and HaCaT A5RT3) (Fig. 1a, b). These findings indicated that increased CCL20 mRNA expression leads to CCL20 protein secretion in these cells. To test whether the observed induction of CCL20 expression is also dependent on EGFR activation, we blocked EGFR signalling in primary human keratinocytes, which exhibit CCL20 expression via autocrine EGFR activation,^16,17^ with the specific tyrosine kinase inhibitor erlotinib,^18–20^ and analysed CCL20 expression after 24 h. In response to EGFR inhibition, we observed a significant reduction in CCL20 expression (Fig. 1c), which corresponded directly to protein levels in the supernatant (Fig. S2A), indicating that CCL20 expression is controlled by the EGFR/Ras signal transduction pathway. In order to validate our results in a broader context, we conducted a systematic search of the NCBI Gene Expression Omnibus (GEO) dataset collection. As shown by Parmenter and colleagues,^21^ CCL20 expression was significantly downregulated in a panel of oncogenic BRAF(V600) mutation-harbouring melanoma cell lines in response to treatment with the BRAF inhibitor vemurafenib (Fig. S3A). Furthermore, a significant decrease in CCL20 levels was shown in murine keratinocytes in response to treatment with thymidine kinase inhibitors erlotinib and PD153036 in Fig. S3B.^22^ In a melanoma cell line carrying NRAS(Q61R) activating mutation,^23^ transfection with NRAS-targeting siRNA constructs also resulted in CCL20 downregulation (Fig. S3C). In vivo tumour samples subjected to single-cell RNA sequencing prior to and after PD-1 inhibitor treatment^24^ also showed a similar tendency; moreover, a marked decrease of CCL20 expression occurred specifically in the cell population identified as malignant melanoma cells (Fig. S3D).^23^ Next, we analysed K5-SOS mice, which express a dominant form of Son of Sevenless (SOS) in the basal keratinocytes. These mice develop EGFR-dependent skin papillomas through the hyperactivation of the RAS pathway within the first few weeks of life.^10^ Indeed, CCL20 expression was elevated in tumour-bearing tail skin of K5-SOS mice as compared with the skin of the respective WT controls (Fig. S4A). In addition, expression of *Ccl20* is significantly downregulated in FACS-purified murine keratinocytes derived from interfollicular epidermis (IFE) of 2-day-old mice lacking EGFR specifically in the epidermal lineage (Fig. S4B). Importantly, these young mice do not present signs of inflammation yet, thus demonstrating a cell-autonomous requirement of EGFR signalling for *Ccl20* expression.^25^ These data indicate that the EGFR/Ras axis augments CCL20 production.

### Tumour cells show enhanced CCL20 production

To extend the findings in the HaCaT cell line to other tumour cell lines, we determined CCL20 expression in established tumour cell lines of breast cancer (*n* = 6), melanoma (*n* = 13) as well as head and neck squamous cell carcinoma (HNSCC) (*n* = 14), compared with the corresponding benign precursor cells, namely human primary mammary epithelial cells, human primary melanocytes and human primary mucosal keratinocytes (Fig. 1d–f). In comparison with mammary epithelial cells, CCL20 expression was higher in MDA-MB-468 and MDA-MB-361, was comparable in MCF-7 and T-47D and was lower in DU-4475 and Hs578T breast cancer cells (Fig. 1d). All analysed melanoma cell lines exhibited higher expression of CCL20 compared with cultured human primary melanocytes, which express CCL20 only at a very low level (Fig. 1e). Within the HNSCC cell lines, half of the analysed lines (UD-SSC 6, UD-SSC 7A, UD-SSC 7B, UM-SSC 10B, UM-SSC 17A and B as well as UT-SSC 24B) expressed higher CCL20 mRNA levels, while the other half exhibited a comparable or slightly lower expression of CCL20 compared with mucosal keratinocytes (Fig. 1f). To correlate the observed CCL20 mRNA levels with protein expression, we investigated CCL20 protein levels in supernatants from HNSCC, melanoma and breast cancer cell lines using ELISA (Fig. S2B). CCL20 was present in all analysed supernatants, indicating that CCL20 mRNA expression corresponds directly to CCL20 protein secretion. Thus, CCL20 mRNA and protein levels are higher in human cell lines representing breast cancer, melanoma and HNSCC compared with the corresponding primary (untransformed) cells.

Next, we investigated CCL20 mRNA expression in tumour biopsies taken from patients suffering from breast cancer, melanoma and HNSCC. We observed significantly higher expression of CCL20 in breast cancer metastases compared with healthy mammary tissue (Fig. 1g). In comparison with melanocytes, primary melanoma subcutaneous and distant metastases all showed significantly increased CCL20 expression (Fig. 1g). In HNSCC, significantly increased CCL20 expression was observed in primary tumour tissue compared with normal mucosa (Fig. 1g). In conclusion, we observed robust enhanced expression of CCL20 in the majority of tested cancer cell lines and tumour samples compared with the corresponding benign precursor cells.

### Tumour-derived CCL20 correlates with ERK activation, advanced cancer stage and poor prognosis

**Fig. 2:**
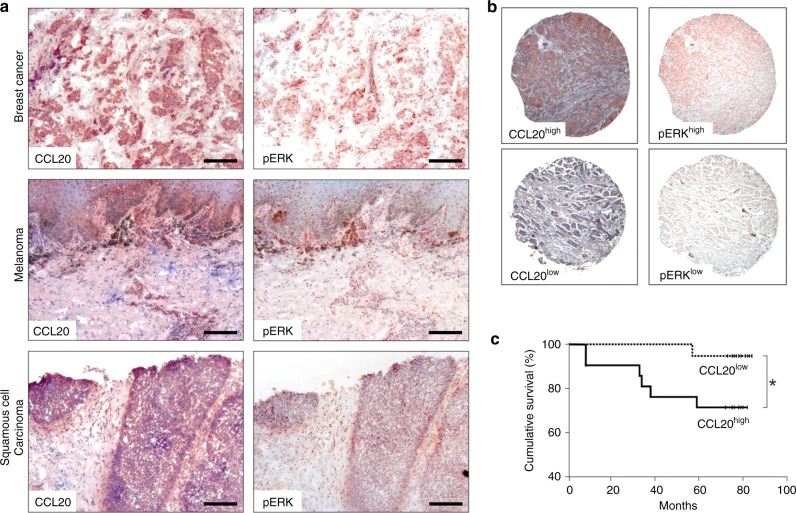
Tumour-derived CCL20 production co-localises to areas of ERK activation and correlates with progressive states of cancer. **a** Representative results of serial sections of breast cancer (*n* = 6), melanoma (*n* = 6) and HNSCC (*n* = 6) stained with anti-CCL20 or anti-pERK1/2 antibodies are shown [scale bars represent 200 µm]. **b** Representative high and low expression of CCL20 and pERK in tumour tissue microarrays. Classifications of CCL20^high^, CCL20^low^, pERK^high^ and pERK^low^ were used for further statistical evaluation of tumour tissue microarrays. **c** Kaplan–Meier graph showing cumulative survival of breast cancer patients as a percentage to follow-up time in months. The follow-up time in this series was calculated from the date of primary tumour excision. Clinical records and follow-up times were obtained from all patients with a median observation time of 77 months (range 9–84 months). Statistical analyses were performed using the Chi-Square test and SPSS software.

As tumour tissues express CCL20 at the mRNA level (Fig. 1g) and EGFR/Ras signalling upregulates CCL20 expression (Fig. 1a–c), we next performed immunohistochemical analysis of CCL20 protein expression and ERK activation in serial sections of tumour samples from breast cancer, melanoma and HNSCC patients (Fig. 2a). Interestingly, sites of strong ERK activation corresponded to sites of strong CCL20 expression (Fig. 2a). For a more extensive approach, we investigated further samples using tumour tissue microarrays of breast cancer, HNSCC and colon carcinoma tumours. CCL20 expression and the presence of pERK were again analysed by immunohistochemical staining (Fig. 2b). The samples were categorised as expressing CCL20 at either high (CCL20^high^) or low levels (CCL20^low^), and also whether they exhibited high activation of ERK (pERK^high^) or low ERK activation (pERK^low^). In 121 cases of ductal breast cancer and HNSCC, we observed high ERK phosphorylation levels in 84.5% of CCL20^high^ tumours, but in only 51% of CCL20^low^-expressing tumours (Table 1). These data therefore show that ERK activation significantly correlates with CCL20 levels (log-likelihood ratio = 12.94, *p* value < 0.0003).

**Table 1:** Correlation of CCL20 and ERK activation and cancer stage.

Correlation of CCL20 and pERK expression				
	# of cases*	pERK^low^ [%]	pERK^high^ [%]	*P* value
Total	121			<0.0003
CCL20^low^ expression	63	49.2	50.8	
CCL20^high^ expression	58	15.5	84.5	

Logistic regression based on CCL20^high/low^ and pERK^high/low^ dichotomisation indicates high predictive value of ERK activation for CCL20 expression levels (log-likelihood ratio = 12.94, *p* value < 0.0003).

*In total, 73 breast cancer tissues and 48 HNSCC tissues were analysed for CCL20 expression and ERK activation. Logistic regression was applied to determine the predictive value between CCL20 and pERK expression.

We also examined CCL20 expression in relation to tumour grade. In 334 cases of breast cancer, colon carcinoma and HNSCC, a higher pT category of the observed tumours correlated significantly to higher expression of CCL20 (Table 2). CCL20 expression and pN category showed a less pronounced correlation, which nevertheless was still statistically significant (Table 2). Taken together, these data demonstrate that CCL20 expression correlates to ERK activation, and that tumours expressing high levels of CCL20 have a more aggressive phenotype.

**Table 2:** Correlation of tumour entity and CCL20 expression.

	# of cases*	CCL20^low^ [%]	CCL20^high^ [%]	*P* value
Total	334			0.004
pT1	15	46.7	53.3	
pT2	111	31.5	68.5	
pT3	157	16.6	83.4	
pT4	51	31.4	68.6	
Total	338			0.048
pN0	219	27.9	72.1	
pN1	64	15.6	84.4	
pN2	50	20.0	80.0	
pN3	5	60.0	40.0	

*Tissue microarrays of colon carcinoma (*n* = 213), breast cancer (*n* = 73) and HNSCC (*n* = 48) were analyzed for CCL20 expression. Expression data were correlated to TNM values (T = tumour size, *N* = degree of spread to regional lymph nodes) and statistically analyzed by Pearson’s Chi-Square test.

Next, we analysed the expression of CCL20 in a breast cancer tumour tissue microarray containing tumour tissues from 40 different patients suffering from ductal breast cancer for which follow-up data were available. Cumulative survival in the CCL20^low^ group after 80 months was 93%, while cumulative survival in the CCL20^high^ group was significantly lower at 70% (Fig. 2c). Thus, high CCL20 expression in primary breast tumours significantly reduces cumulative survival of the patients after tumour excision. Together, our results suggest that tumour-derived CCL20 promotes tumour progression and growth, and has a corresponding negative effect on the survival of breast cancer patients.

### Endothelial cells express the CCL20-specific chemokine receptor CCR6

**Fig. 3:**
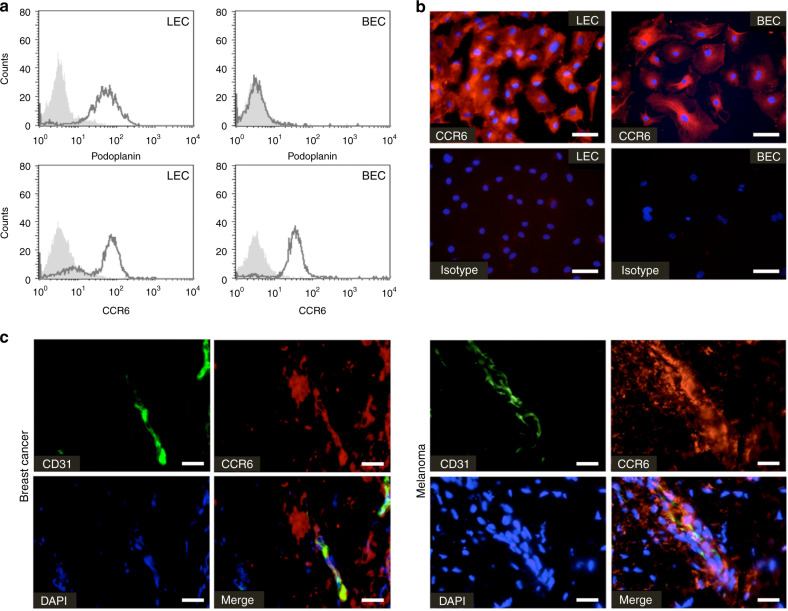
Microvascular endothelial cells express CCR6 on their cell surface. **a** Flow cytometric analysis of podoplanin and CCR6 protein surface expression in podoplanin-positive lymphatic microvascular endothelial cells (LEC) or podoplanin-negative blood microvascular endothelial cells (BEC) (black line). Filled histogram shows isotype control. **b** Immunofluorescence analysis of CCR6 (red) expression of LECs and BECs [scale bars represent 50 µm]. **c** Immunofluorescence analysis of CD31 (green) and CCR6 (red) expression in marginal zones of a breast cancer tumour and melanoma demonstrates the co-localisation of CCR6 with microvessels in the tumour microenvironment [scale bars represent 25 µm]. CCR6 seems also expressed by cancer cells, stromal cells and immune cells infiltrating the tumour.

We next aimed at unravelling the underlying mechanism of tumour-derived CCL20 promotion of tumour progression, growth and poor prognosis. We hypothesised that tumour-derived CCL20 creates a microenvironment that enhances tumour growth. Given that the vasculature plays a major role in regulating tumour growth as well as metastasis, we investigated whether microvascular endothelial cells express CCR6, the corresponding receptor for CCL20, on their surface. In FACS analyses, abundant CCR6 expression was observed on podoplanin-negative human blood microvascular endothelial cells (BEC) as well as podoplanin-positive human lymphatic microvascular endothelial cells (LEC) (Fig. 3a). Immunofluorescence analyses confirmed strong cell surface expression of CCR6 on microvascular endothelial cells, and also indicated the presence of a cytoplasmatic pool of CCR6 (Fig. 3b). Furthermore, we found that vessels in the vicinity of CCL20-positive tumour tissues expressed CCR6. Tissue sections of breast cancer and melanoma were double-stained for CD31 and CCR6 using immunofluorescence. CD31/CCR6-positive vessels were found associated with tumours of breast cancer and melanoma patients (Fig. 3c). The fact that CCR6-positive vessels are closely apposed to CCL20-expressing tumours allowed us to hypothesise that CCL20 might be able to functionally activate the microvasculature and induce angiogenesis.

### CCL20 promotes endothelial cell migration, tube formation and angiogenesis

**Fig. 4:**
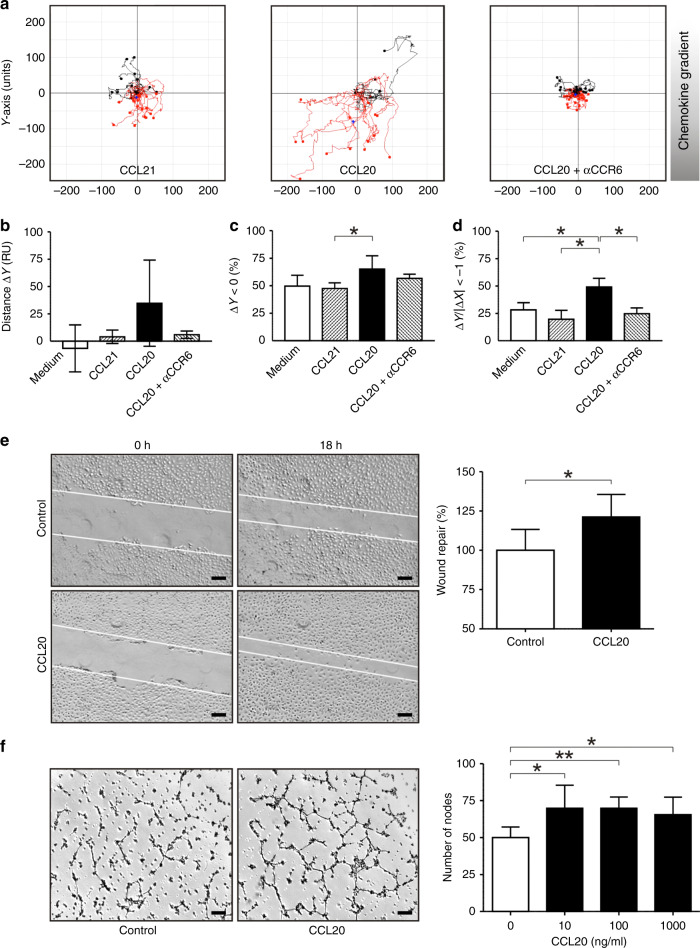
CCL20 mediates directional migration of human microvascular endothelial cells and enhances tube formation. **a** Representative trajectories of microvascular endothelial cells cultured inside an IBIDI µ-chemotaxis chamber containing CCL20 (1000 ng/ml), CCL21 (1000 ng/ml; “irrelevant” (the corresponding receptor is absent on the target cell) chemokine control) or endothelial cells pre-incubated with anti-CCR6 antibodies and cultured in slides containing CCL20 (1000 ng/ml). Trajectories are representatives for test and control stimuli of at least three different experiments. **b**–**d** Statistical analysis of endothelial cell trajectories in chemotaxis chambers. **b** Average ∆*Y*, mean net distance (RU) travelled along the chemokine gradient (*Y* axis). **c** ∆*Y* < 0, percentages of endothelial cells travelling in the direction of chemokine gradients (*Y* axis). (**d**) ∆*Y*/I∆*X*I < −1, percentages of endothelial cells travelling a longer distance in the direction of chemokine gradients (*Y* axis) than in the direction orthogonal to the gradients (*X* axis) (*P ≤ 0.05, Mann–Whitney *U* test). **e** In monolayer wound-repair assays, CCL20 was able to induce significant wound repair and migration of microvascular endothelial cells when compared with corresponding controls (*P ≤ 0.05, Mann–Whitney *U* test) [scale bars represent 200 µm]. **f** Tube-formation assay with microvascular endothelial cells showing representative images of endothelial cell tube formation on Matrigel alone or Matrigel supplemented with CCL20 (10 ng/ml), and statistical analysis of tube-formation assays. Number of nodes per field are shown and represent the mean ± SD of three independent experiments (*P ≤ 0.05; **P ≤ 0.01, Mann–Whitney *U* test) [scale bars represent 200 µm].

To test our hypothesis that tumour-derived CCL20 might participate in tumour angiogenesis, we investigated the effect of CCL20 on microvascular endothelial cells in vitro. Chemotaxis assays were performed using IBIDI µ-chemotaxis slides. The strongest chemotactic response was observed with CCL20 gradients, while response rates obtained with the irrelevant (corresponding receptor is absent on the target cell) chemokine CCL21 or with medium were equivalent to values expected for randomly moving cells showing no chemotaxis (Fig. 4a–d). Furthermore, chemotaxis of BEC towards CCL20 was significantly impaired using neutralising anti-human CCR6 antibodies, suggesting an important role for CCL20–CCR6 interactions in guiding BEC. The motility-enhancing effect of CCL20 was further substantiated in monolayer wound-repair assays. At doses of 100 ng/ml, CCL20 induced faster monolayer wound closure than control medium alone (Fig. 4e).

Another facet of the angiogenic response is the induction of capillary tube formation by endothelial cells. The ability of CCL20 to influence tube formation was assayed in vitro by plating endothelial cells on Matrigel and treating them with varying concentrations of CCL20 (10–1000 ng/ml), or with PBS as a control. Three independent experiments were performed, and the data were analysed by counting nodes of three or more tubes. In the control, 50 (SD ± 7) nodes were observed. Significantly more nodes were induced by CCL20, as 10 ng/ml CCL20 induced 70 (SD ± 16), 100 ng/ml CCL20 induced 70 (SD ± 8) and 1000 ng/ml CCL20 induced 66 (SD ± 12) nodes per well (Fig. 4f). We therefore conclude that CCL20 is able to promote endothelial cell angiogenesis.

### CCR6 deficiency of the host results in impaired tumour-associated angiogenesis and tumour progression

**Fig. 5:**
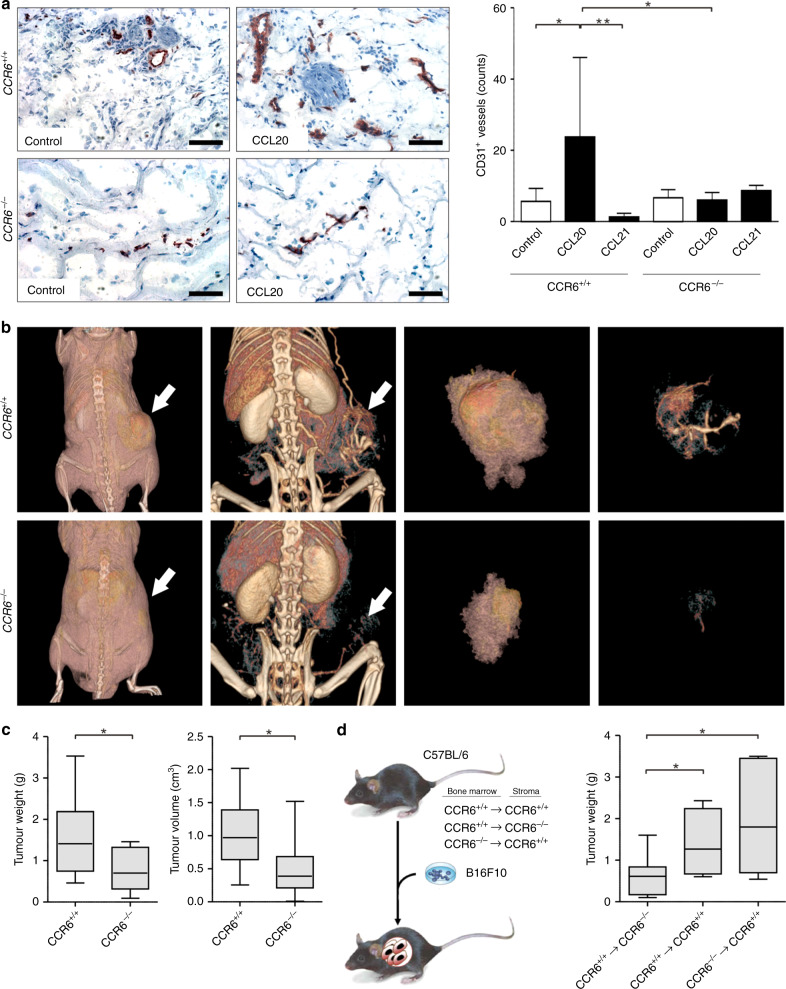
CCL20/CCR6 signalling supports angiogenesis in vivo. **a** Cross sections of Matrigel plugs removed 21 days after injection into C57BL/6 mice were stained with anti-CD31 antibodies [scale bars represent 100 µm]. Representative pictures and the number of CD31-positive vessels per cross section are shown and represent the mean ± SD of three independent experiments (*P ≤ 0.05; **P≤0.01, Mann–Whitney U test). **b** Left panels: FpVCT scans showing contrast agent-containing tumour vessels and their distribution and bifurcations in the periphery and within CCL20-expressing B16F10 tumours growing in C57BL/6 wild-type and C57BL/6-*CCR6*^–/–^ mice. Representative results are shown. Tumours are indicated by white arrows. Right panels: evaluation of the weight and volume of B16F10 tumours in C57BL/6 wild-type and C57BL/6-*CCR6*^–/–^ mice. Tumour weight was measured in grams. The data represent the mean ± SD of twelve independent tumours. Tumour volume was automatically determined by fpVCT datasets and measured in cm^3^. The data represent the mean ± of eight independent tumours (*P ≤ 0.05, Mann–Whitney *U* test). **c** Scheme showing the different bone marrow chimeras generated for tumour experiments. **d** Analysis of B16F10 tumours in bone marrow chimeras. Tumour weight was measured in grams. Data represent the mean ± SD of seven independent tumours (*P ≤ 0.05, Mann–Whitney *U* test).

To determine whether CCL20 influences angiogenesis in vivo, we examined the effect of CCL20 on the growth of blood capillaries in subcutaneous Matrigel plugs. Plugs consisting of Matrigel alone, as well as plugs containing murine Ccl20 and human CCL21, were injected into C57BL/6 and CCR6-deficient C57BL/6 mice. Human CCL21 was used as a negative control. Endothelial cells express CXCR3 on their cell surface, and murine Ccl21 therefore exerts an angiostatic effect.^26^ Human CCL21, on the other hand, has been demonstrated to bind to murine Ccr7 but not to murine Cxcr3,^27^ and therefore represents a suitable control for this experiment. After excision, plugs were analysed for the number of CD31-positive vessels (Fig. 5a). In control plugs from wild-type and Ccr6-deficient mice, few vessels were observed (mean 5.6 vessels/field; SD ± 3.6 in wild type, mean 6.6 vessels/field; SD ± 5.1 in Ccr6-deficient) (*p* = 0.6688, Mann–Whitney *U* test) (Fig. 5a). In Matrigel plugs containing 1000 ng/ml Ccl20, many vessels were observed after growth in wild-type mice (mean 23.8 vessels/field; SD ± 22.2) (*p* = 0.019, Mann–Whitney *U* test, compared with wild-type control plugs), whereas in Ccr6-deficient mice, significantly fewer vessels were present (mean 6.1 vessels/field; SD ± 4.7) (*p* = 0.0426, Mann–Whitney *U* test). Importantly, in plugs from Ccr6-deficient mice, the number of vessels in control plugs was comparable to that in the Ccl20-containing plugs (*p* = 0.6548, Mann–Whitney *U* test). These data therefore demonstrate that Ccl20 is able to recruit Ccr6-positive vessels. As a further control, CCL21 was included in Matrigel plugs. In C57BL/6 mice, vessel density was 1.3 vessels/field (SD ± 2.1) (*p* = 0.009, Mann–Whitney *U* test, compared with CCL20 plugs in wild-type mice), whereas in Ccr6-deficient mice, the number of vessels was 8.7 vessels/field (SD ± 3.4) (*p* = 0.8766, Mann–Whitney *U* test, compared with CCL20 plugs in Ccr6-deficient mice). This result demonstrates that vessel recruitment was specific to the CCL20/CCR6 interaction and not a general effect of supplying chemokines in Matrigel. The observation that Ccl20 was able to promote vessel formation in the Matrigel plugs suggests that, in the tumour microenvironment, CCL20 may be able to induce and/or enhance tumour angiogenesis.

Next, we investigated whether CCL20 influences tumour vascularisation. B16/F10 tumour cells, which express Ccl20, were subcutaneously injected into the right flank of wild-type and Ccr6-deficient C57BL/6 mice. Two weeks after injection of the cells, the vasculature inside the resulting tumours was imaged by flat-panel volume computed tomography (fpVCT) to allow three-dimensional (3D) visualisation of anatomical structures (Fig. 5b). Analysis of differences in tumour and vessel growth between wild-type and Ccr6-deficient mice revealed that subcutaneous B16/F10 tumours in wild-type mice recruited a dense network of blood vessels (Fig. 5b, white arrows), while tumours in Ccr6-deficient mice showed dramatically fewer tumour-infiltrating vessels (Fig. 5b). Not only was the vascularisation rate of tumours in wild-type mice increased, but also the number, size and diameter of the vessels were larger. In addition, volume analysis of fpVCT datasets demonstrated that tumour volumes were significantly larger in wild-type mice than in Ccr6-deficient mice (Fig. 5b). At the end of the experiment, autopsies were performed, and the tumours were excised and weighed (Fig. 5b). Tumours grown in Ccr6-deficient mice (0.76 g ± 0.49 g) had a significantly reduced weight compared with tumours from wild-type mice (1.38 g ± 0.76 g). Furthermore, immunohistochemical analysis of CD31-positive vessels in tumour sections revealed that there were fewer microvascular and macrovascular vessels inside the tumours from Ccr6-deficient mice (10 ± 8.95) compared with the tumours from wild-type mice (26 ± 21.7) (Fig. S5).

CCL20 is known to act as a chemoattractant for CCR6 expression bone marrow-derived cells, and to recruit inflammatory cells, e.g. dendritic cells and regulatory T cells to sites of cancer.^28,29^ CCL20 could therefore in principle affect tumour growth through effects on bone marrow-derived immune cells rather than through angiogenesis. To determine if this is the case, bone marrow chimeras were generated bearing either a wild-type or Ccr6-deficient immune system, while retaining a wild-type stroma (Ccr6^+/+^ → Ccr6^+/+^ and Ccr6^−/−^ → Ccr6^+/+^). In addition, a chimera bearing a CCR6-deficient stroma, but a wild-type immune system, was generated (Ccr6^+/+^ → Ccr6^–/–^) (Fig. 5c). Again, B16F10-tumour cells were subcutaneously injected into the chimeras as described above and analysed after 22 days. The tumours in Ccr6^+/+^ → Ccr6^+/+^ (1.27 g ± 0.82 g) and Ccr6^−/−^ → Ccr6^+/+^ (1.8 g ± 1.25 g) mice showed comparative weights, whereas Ccr6^+/+^ → Ccr6^−/−^ (0.61 g ± 0.48 g) mice presented with tumours of significantly lower weight comparable to Ccr6-deficient C57BL/6 mice (Fig. 5d). Together, these data demonstrate that expression of the chemokine receptor CCR6 in the tumour microenvironment promotes tumour growth and is important for efficient recruitment of vessels into the tumour, and that this phenotype is dependent on stromal expression of CCR6 and not on expression of the receptor in cells of the immune system.

## Discussion

Emerging evidence indicates that cancer development, progression and metastasis is not a cancer cell-autonomous process, but rather dependent on complex multicellular interactions within a newly formed tissue, the cancer tissue.^30^ The concept of the combined effect of different cell types (tumour cells, endothelial cells, fibroblasts, pericytes and immune cells), the matrix and soluble factors was coined the tumour microenvironment.^31,32^ Tumour cells drive the establishment of their microenvironment by secretion of cytokines and chemokines, which act on the surrounding stroma cells and infiltrating immune cells, which in turn provide factors supporting tumour growth and progression. Here we show that EGFR/Ras signalling in tumour cells induces the production of CCL20 in a variety of types of cancers. Moreover, we show that CCL20 stimulates angiogenesis, which fosters tumour growth and progression.

The activation of several signal transduction pathways, among them the EGFR/Ras/MAPK pathway, is responsible for switching cells from a resting phenotype into a proliferative and migratory one. Earlier, we investigated the effect of activation of the Ras/MAPK pathway in cutaneous skin cancer, and observed a loss of homoeostatic chemokine expression.^6^ In our investigation of chemokines upregulated by Ras/MAPK activation in HaCaT keratinocytes, we observed a significant upregulation of the chemokine CCL20, surpassing the expression of the known EGFR/Ras-inducible chemokine CXCL8.^33^ Consistently, we have also recently demonstrated that pharmacologic inhibition of the EGFR significantly downmodulated the expression of CCL20 and CCL8.^34^ The expression of CCL20 shows variability between the individual tumour cell lines. This likely correlates with the activation levels of the RAS/RAF/MAPK pathway. For instance, breast cancer RAS pathway activation is found in most widely studied human breast cancer cell lines.^35^ Eckert et al. investigated RAS activation of MDA-MB-468 and T-47D cell lines. In this study, RAS activation was much higher in MDA-MB-468 cells compared with T-47D cells,^35^ which correlates with CCL20 expression in our study. In cancer tissues, including breast cancer, colon carcinoma, head and neck SCC and melanoma, our results indicate that CCL20 is a chemokine preferably expressed in advanced tumour stages. In addition, in breast cancer patients, we observed a significant correlation between high CCL20 expression and adverse survival rates. In recent findings, elevated CCL20 expression was also observed in several other cancer types, e.g. pancreatic cancer, non-small- cell lung cancer, hepatocellular carcinoma and glioma.^36–39^ In pancreatic cancers, it has been suggested that CCL20 contributes to the promotion of transformed cell growth by modulation of diverse tumour cell functions such as stroma dissolution and migration.^40^ Furthermore, it has been shown that the interaction between CCL20 and CCR6 leads to chemokine-mediated regulatory T-cell recruitment.^41^ Several studies have reported that the more regulatory T cells infiltrate to tumour sites, the worse prognosis of cancer patients.^42^ Therefore, the expression of CCL20 in diverse cancer entities hints at a general role for CCL20 in the progression of carcinomas, irrespective of the tissues in which these cancers arise, and thereby might be an important factor in establishing a microenvironment driving a more aggressive and fatal disease. Our systematic database search of CCL20 gene expression in multiple cell types harbouring a variety of oncogenic mutations also revealed a negative correlation between CCL20 levels and treatment response.

Our analysis of possible targets for CCL20 action within the tumour microenvironment implicated microvascular endothelial cells due to their high expression of CCR6 on their cell surface in vitro, and due to the observation of CD31^+^/CCR6^+^ vessels closely apposed to tumour tissue of patients. Indeed, the results of our experiments analysing the functional effect of CCL20 in vitro revealed a migratory and angiogenic effect of CCL20 on endothelial cells. The recruitment of CD31^+^ vessels into Matrigel plugs supplied with Ccl20 and injected into mice, which was abrogated when plugs were injected into a Ccr6-deficient mouse strain, further substantiates an angiogenic role for CCL20 in the tumour microenvironment.

Chemokines have been shown to exhibit angiogenic and angiostatic properties.^43^ The CXCR2 receptor mainly transduces angiogenic signals.^44^ Accordingly, the chemokines CXCL8, CXCL1–3 and CXCL6, which all bind to CXCR2, are active inducers of angiogenesis.^43^ In particular, Strieter and co-workers showed that CXCL8 is angiogenic and plays a role in tumour-associated angiogenesis of non-small-cell lung cancer (NSCLC).^45^ Only recently, CCL20 and its role in angiogenesis in hepatitis C infection was reported by an independent group.^46^ In wild- type C57BL/6 mice, tumours derived from B16F10 cells contained a dense vascular network that included large, mature vessels within the tumour. In contrast, B16F10 tumours grown in Ccr6-deficient mice displayed dramatically reduced numbers of tumour-infiltrating vessels, and were notable for their lack of a macrovasculature. In addition, tumours in Ccr6-deficient mice were smaller in volume and had less weight than tumours grown in a wild-type background, therefore confirming our in vitro findings. How do these results fit with previously known functions of CCL20? As CCL20 has long been known to play a role in immature dendritic cell recruitment.^47^ Recent studies have also shown that regulatory T cells are recruited into the tumour micromilieu via the CCL20/CCR6 axis.^48^ Therefore, CCL20 expression in tumours could conceivably influence the antitumour immune response. We therefore examined the importance of CCR6 expression in bone marrow-derived immune cells for the observed phenotype. To this end, we examined B16F10-tumour growth in bone marrow chimeras harbouring either a Ccr6-deficient immune system in a wild-type host or vice versa. Interestingly, after 17 days, Ccr6^+/+^ → Ccr6^–/–^ mice presented with a significantly lower tumour growth and vascularisation of the tumour than tumours grown in Ccr6^+/+^ → Ccr6^+/+^ control mice. In contrast, B16F10 tumours grown in Ccr6^−/−^ → Ccr6^+/+^ mice were not significantly larger than tumours grown in a Ccr6^+/+^ → Ccr6^+/+^ mice background. Thus, a Ccr6-positive immune system did not significantly affect tumour growth, indicating that the action of CCL20 on angiogenesis is more important during tumour progression than its role in immune cell recruitment in this model. This notion is further corroborated by the observations in patients, where high CCL20 expression correlated with larger and more advanced tumours. Thus, we conclude that the main role for expression of CCL20 in the tumour microenvironment is to stimulate recruitment of vessels into the growing tumour mass, resulting in faster growth and progression of the tumour. However, the role of CCL20 may differ among cancer entities. Especially in immunogenic tumours, the recruitment of infiltrating regulatory T cells by CCL20 expression should be additionally considered.

In summary, we demonstrate that EGFR/Ras-induced CCL20 is a chemokine with a vital function in tumour–stroma interaction within the tumour micromilieu. CCL20 is a component of the complex regulation of tumour-associated angiogenesis, and therefore its receptor CCR6 might represent a promising target for anticancer therapy. Specifically, blocking the activity of CCR6 in the microenvironment of the tumour might inhibit tumour neoangiogenesis and thereby enhance conventional antitumour therapies.

## Supplementary information

Supplementary Material

## Data Availability

All data and materials can be found in the material section or can be accessed via B.H.
