# Correction to: EGFR/Ras-induced CCL20 production modulates the tumour microenvironment

**DOI:** 10.1038/s41416-021-01563-y

**Published:** 2021-10-01

**Authors:** Andreas Hippe, Stephan Alexander Braun, Péter Oláh, Peter Arne Gerber, Anne Schorr, Stephan Seeliger, Stephanie Holtz, Katharina Jannasch, Andor Pivarcsi, Bettina Buhren, Holger Schrumpf, Andreas Kislat, Erich Bünemann, Martin Steinhoff, Jens Fischer, Sérgio A. Lira, Petra Boukamp, Peter Hevezi, Nikolas Hendrik Stoecklein, Thomas Hoffmann, Frauke Alves, Jonathan Sleeman, Thomas Bauer, Jörg Klufa, Nicole Amberg, Maria Sibilia, Albert Zlotnik, Anja Müller-Homey, Bernhard Homey

**Affiliations:** 1 Department of Dermatology, Medical Faculty, Heinrich Heine University, Düsseldorf, Germany; 2 Department of Dermatology, University Hospital Münster, Münster, Germany; 3 Department of Dermatology, Venereology and Oncodermatology, Medical Faculty, University of Pécs, Pécs, Hungary; 4 Department of Pediatric Cardiology and Intensive Care Medicine, Medical Center Georg August University Göttingen, Göttingen, Germany; 5 Clinic of Hematology and Medical Oncology, University Medical Center Göttingen, Göttingen, Germany; 6 Dermatology and Venereology Unit, Department of Medicine, Karolinska Institutet, Stockholm, Sweden; 7 Department of Dermatology and UCD Charles Institute for Dermatology, University College Dublin, Dublin, Ireland; 8 Department of Pharmacology, Medical Faculty, Heinrich Heine University, Düsseldorf, Germany; 9 Precision Immunology Institute, Icahn School of Medicine at Mount Sinai, New York, NY USA; 10 Leibnitz Research Institute for Environmental Medicine, Düsseldorf, Germany; 11 Department of Physiology and Biophysics, University of California, Irvine, CA USA; 12 Department of General-, Visceral- and Pediatric Surgery, Medical Faculty, Heinrich Heine University, Düsseldorf, Germany; 13 Department of Otorhinolaryngology, Head and Neck Surgery, University Medical Center, Ulm, Germany; 14 European Center for Angioscience (ECAS), Medical Faculty of Mannheim, Heidelberg University, Mannheim, Germany; 15 Karlsruhe Institute for Technology (KIT), Campus Nord, Institute for Toxicology and Genetics, Karlsruhe, Germany; 16 Institute of Cancer Research, Department of Medicine I, Medical University of Vienna and Comprehensive Cancer Center, Vienna, Austria; 17 Insitute of Science and Technology Austria, Klosterneuburg, Austria; 18 Insitute for Immunology, University of California, Irvine, CA USA; 19 Department of Radiation Oncology, Medical Faculty, Heinrich Heine University, Düsseldorf, Germany

**Keywords:** Cancer microenvironment, Chemokines

Correction to: *British Journal of Cancer* 10.1038/s41416-020-0943-2, published online 30 June 2020

The article ‘EGFR/Ras-induced CCL20 production modulates the tumour microenvironment’, written by Andreas Hippe, Stephan Alexander Braun, Péter Oláh, Peter Arne Gerber, Anne Schorr, Stephan Seeliger, Stephanie Holtz, Katharina Jannasch, Andor Pivarcsi, Bettina Buhren, Holger Schrumpf, Andreas Kislat, Erich Bünemann, Martin Steinhoff, Jens Fischer, Sérgio A. Lira, Petra Boukamp, Peter Hevezi, Nikolas Hendrik Stoecklein, Thomas Hoffmann, Frauke Alves, Jonathan Sleeman, Thomas Bauer, Jörg Klufa, Nicole Amberg, Maria Sibilia, Albert Zlotnik, Anja Müller-Homey and Bernhard Homey, was originally published electronically on the publisher’s internet portal on 30 June 2020 without open access. With the author(s)’ decision to opt for Open Choice the copyright of the article changed on 16 September 2021 to © The Author(s) 2021 and the article is forthwith distributed under a Creative Commons Attribution 4.0 International License, which permits use, sharing, adaptation, distribution and reproduction in any medium or format, as long as you give appropriate credit to the original author(s) and the source, provide a link to the Creative Commons licence, and indicate if changes were made. The images or other third party material in this article are included in the article’s Creative Commons licence, unless indicated otherwise in a credit line to the material. If material is not included in the article’s Creative Commons licence and your intended use is not permitted by statutory regulation or exceeds the permitted use, you will need to obtain permission directly from the copyright holder. To view a copy of this licence, visit http://creativecommons.org/licenses/by/4.0/.

Open Access funding enabled and organized by Projekt DEAL.

