# Supplementary material for: EGFR/Ras-induced CCL20 production modulates the tumour microenvironment
Source: Br J Cancer. 2020 Jun 30;123(6):942–54. doi: 10.1038/s41416-020-0943-2 (PMC7493992; doi:10.1038/s41416-020-0943-2)
Supplement: Supplementary file 1 — Supplementary Material [file 41416_2020_943_MOESM1_ESM.docx]

**SUPPLEMENTARY MATERIAL**


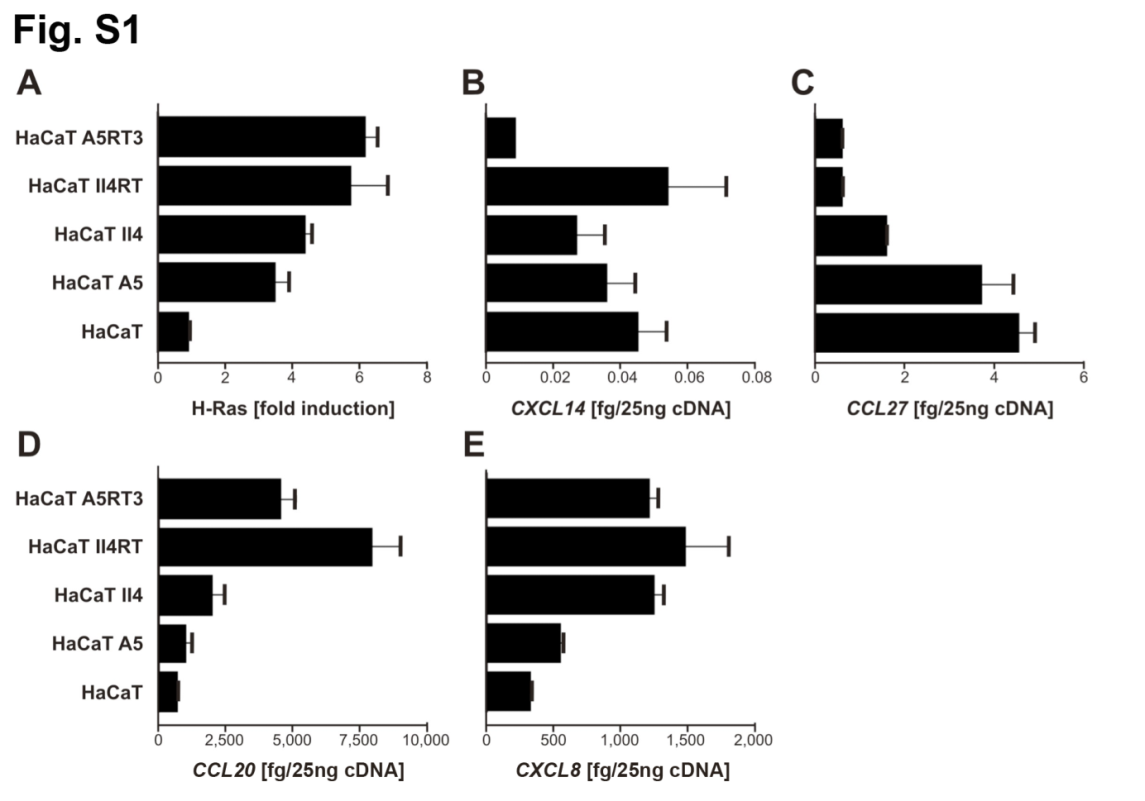


**Figure S1.** **Chemokines are regulated by Ras activation.** (A) Relative Ras activity in the immortalized keratinocyte cell line HaCaT and in H-RasV12-transfected HaCaT clones were assayed by the EZ-Detect Ras activation kit. (B,C) CXCL14 and CCL27 mRNA expression in untransfected HaCaT cells and H-RasV12-transfected HaCaT clones was analyzed by qPCR. Both chemokines show a marked downregulation in correlation to Ras activation. (D,E) CCL20 and CXCL8 mRNA expression in untransfected HaCaT cells and H-RasV12-transfected HaCaT clones. Both chemokines are upregulated by Ras activation. CCL20 shows overall a higher induction than CXCL8. Therefore, CCL20 was chosen for further analysis. Values are expressed as femtograms of target gene per 25 ng of cDNA and represent the mean ± SD of three independent experiments.


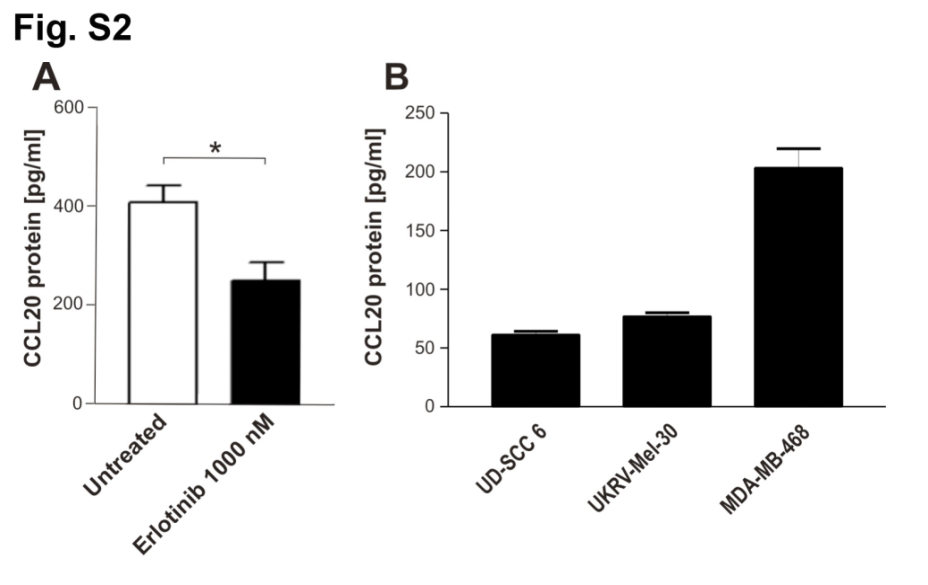


**Figure S2.** **CCL20 protein expression is inhibited by erlotinib treatment of primary keratinocytes**. A) CCL20 protein expression is inhibited by Erlotinib treatment of primary keratinocytes. Activated primary keratinocytes were treated with the selective irreversible inhibitor of EGFR tyrosine kinase Erlotinib and expression of CCL20 was analyzed by a specific ELISA system in supernatants of cell culture. (B) Tumor cell lines secrete CCL20 protein. CCL20 protein expression was measured in supernatants of HNSCC (UD-SCC6), melanoma (UKRV-Mel-30) and breast cancer (MDA-MB-468) cell lines using a specific ELISA system.


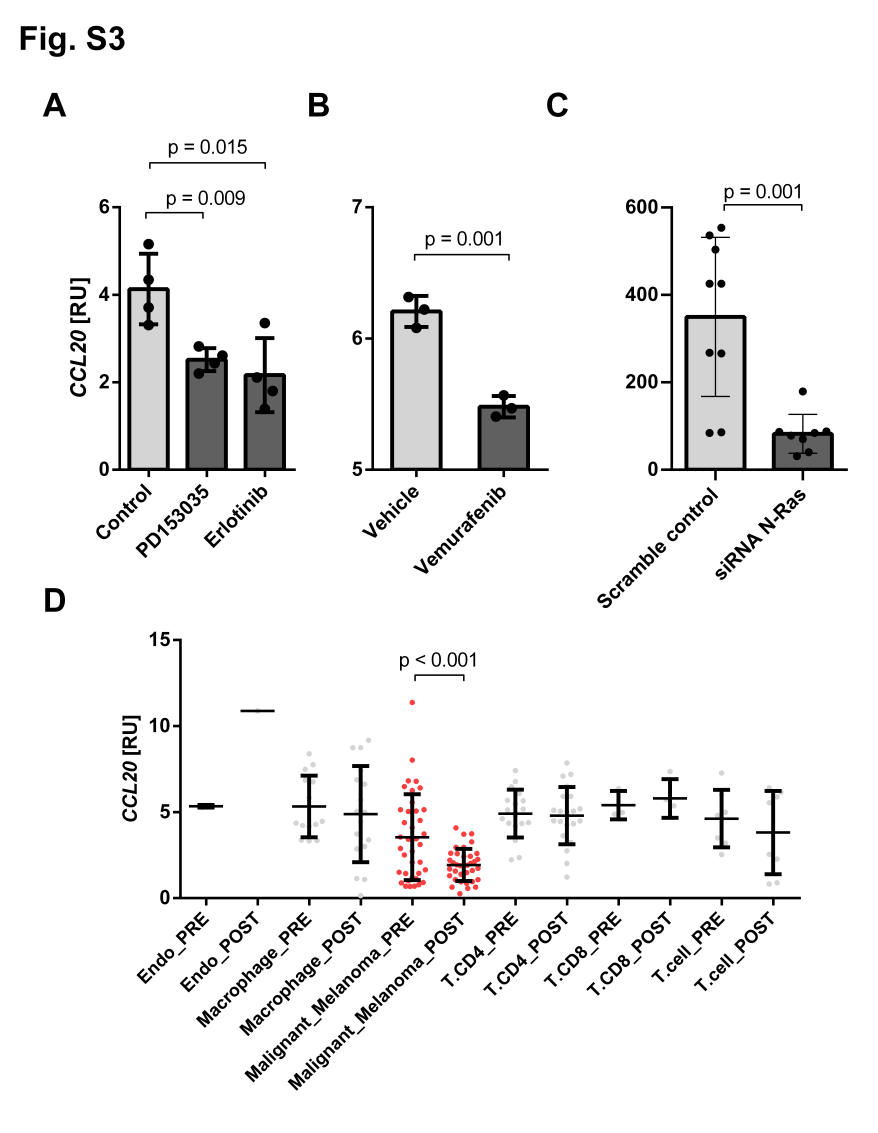


**Figure S3. CCL20 gene expression in publicly available tumor datasets.** (A) Effect of Vemurafenib treatment on CCL20 expression in oncogenic BRAF harbouring melanoma cell line. Normalized expression data reanalysed from Parmenter et al.^21^. (B) Expression of CCL20 in murine keratinocytes treated with Erlotinib or PD153036 TK inhibitor, respectively. Normalized expression data reanalysed from Wright et al.^22^. (C) CCL20 expression in melanoma cell lines treated with siRNA construct targeting NRAS(Q61R) compared to siRNA-scramble controls. Normalized expression data reanalysed from Eskandarpour et al.^23^. (D) Single-cell RNA-sequencing data^24^ were reanalysed and CCL20 expression extracted per distinct cell types, before and during anti-PD1 therapy. Malignant melanoma cells exhibited significantly decreased levels of CCL20 post-treatment (highlighted in red). Rel. Norm. Exprs.: relative normalized expression as defined in original GEO datasets.

**
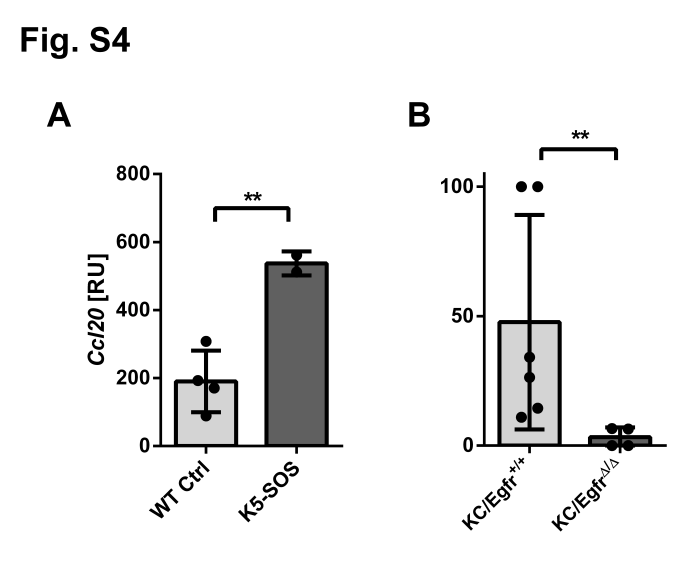
**

**Figure S4. CCL20 gene expression in murine models.** (A) Expression of *Ccl20* in the tail skin of WT and tumour bearing *K5-SOS* transgenic mice, as measured by qPCR (p-value=0.007). Bars indicate relative expression +/- SD. (B) *Ccl20* expression in FACS-sorted *EGFR* wild-type and *K5-Cre* conditional knock-out (KO) murine keratinocytes (p-value=0.002).


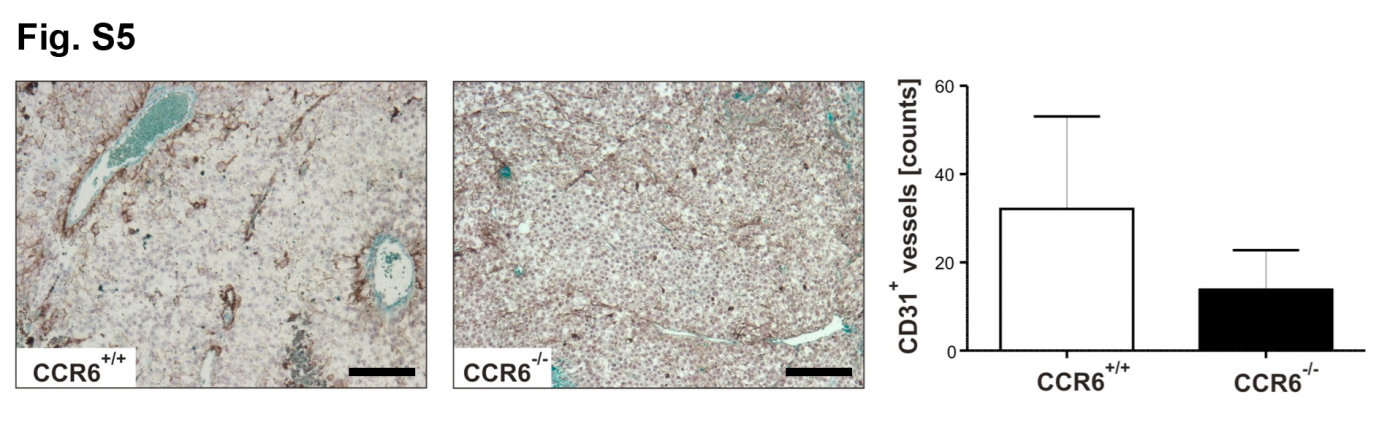


**Figure S5. CD31-positive vessel density is lower in B16F10 tumors of C57BL/6-CCR6-/- mice compared to tumors grown in C57BL/6 mice.** Analysis of CD31-positive vessels in B16F10-tumors of C57BL/6 wildtype and C57BL/6-CCR6-/- mice. Slides were stained with specific antibodies and number of vessels counted in three ROI of tumors (n=9) [scale bars represent 200 µm].
